# Synergies between Communicable and Noncommunicable Disease Programs to Enhance Global Health Security

**DOI:** 10.3201/eid2313.170581

**Published:** 2017-12

**Authors:** Deliana Kostova, Muhammad J. Husain, David Sugerman, Yuling Hong, Mona Saraiya, Jennifer Keltz, Samira Asma

**Affiliations:** Centers for Disease Control and Prevention, Atlanta, Georgia, USA

**Keywords:** global health security, emergency preparedness, noncommunicable diseases, communicable diseases, disease programs, synergies

## Abstract

Noncommunicable diseases are the leading cause of death and disability worldwide. Initiatives that advance the prevention and control of noncommunicable diseases support the goals of global health security in several ways. First, in addressing health needs that typically require long-term care, these programs can strengthen health delivery and health monitoring systems, which can serve as necessary platforms for emergency preparedness in low-resource environments. Second, by improving population health, the programs might help to reduce susceptibility to infectious outbreaks. Finally, in aiming to reduce the economic burden associated with premature illness and death from noncommunicable diseases, these initiatives contribute to the objectives of international development, thereby helping to improve overall country capacity for emergency response.

The first cohort of infants with Zika virus–related birth defects was reported in 2015 in Brazil, where >4,000 cases of infant microcephaly were documented by the end of that year (*1*). Brazil’s Zika outbreak was a recent occurrence, but the country’s baseline preparedness for public-health disruptions has had a relatively long history. The foundation for the emergency response to the 2015 epidemic can be traced back to 1988, when Brazil introduced a health services system for the provision of primary and prenatal care (*2*). As the spread of Zika intensified in 2015, this system provided the infrastructure for recognizing and handling the epidemic relatively quickly. No Zika transmission occurred during the 2016 Summer Olympics in Brazil, despite increased international travel to and from the country at that time. The relatively quick detection of Zika enabled by Brazil’s primary health network and health surveillance system may have increased the ability to control the epidemic at the source, ultimately enhancing global health security.

Brazil’s experience with Zika has illustrated the interplay between 2 key factors that determine the strength of global health security: 1) country capacity for rapid response to emerging contagions (emergency preparedness), and 2) the strength of ongoing activities to support the underlying population health (health infrastructure). In this example, the interconnectedness between these 2 factors was highlighted by the contribution of the existing primary care infrastructure to the success of the Zika emergency response. Brazil’s public health system, ordinarily set up for routine care rather than emergent outbreak containment, enabled the documentation of new cases of the not-yet-identified infection, and an existing surveillance platform, the Notifiable Diseases Information System, facilitated identification of the epidemic by tracking confirmed Zika cases and their birth defect consequences (*1*). These preexisting structures, which typically address common health needs such as maternal and noncommunicable disease (NCD) care, might have been instrumental in ensuring a timely response to Zika, potentially helping to reduce the risk of cross-border spread of the virus. By contrast, such structures were not present during the 2014 outbreak of Ebola in West Africa, where the virus affected multiple countries and threatened to spread to other continents before being contained at high financial and human cost.

## Role of NCD Prevention and Control in Global Health Security

NCDs, represented primarily by cardiovascular disease, cancer, chronic respiratory disease, and diabetes, have overtaken communicable diseases as the leading sources of premature death and disability in low- and middle-income countries (LMICs) (*3*). During 1990–2010, low-income countries experienced a 42% increase in NCD-related death and disability while sustaining a 14% decline in communicable disease burden (*4*).

As NCDs become the leading disease category in developing countries, the provision of NCD-related services increasingly forms the backbone of health delivery systems, helping to build up infrastructures that would be essential for containing infectious disease emergencies. This fact means that it is no longer fitting to consider global health security from the perspective of infectious disease alone. NCD initiatives—programs that support the prevention and control of NCDs—are essential for global health security in several ways. First, in addressing health needs that typically require long-term chronic care, these programs support the development of stable health delivery and health monitoring systems, which can serve as necessary platforms for emergency preparedness in low-resource environments. Second, by improving the baseline level of population health, the programs might help to reduce susceptibility to infectious outbreaks. Finally, in aiming to reduce economic pressures associated with premature illness and death from NCDs in LMICs, these initiatives contribute to the goals of international development, thereby helping to improve overall country capacity for emergency response.

### NCD Initiatives as a Means for Improving Health System Capacity

As demonstrated by Brazil’s experience with Zika, a health delivery system that ordinarily addresses NCDs can be a first line of defense when a communicable disease emergency occurs. Integration between initiatives to reduce NCDs and communicable diseases has been recognized as essential for efficient distribution and use of health resources, both human and financial (*5*), and can provide a sustainable foundation for emergency preparedness (*6*). Specifically, in the context of developing countries, a health infrastructure built around routine and/or long-term NCD services can provide crucial support for emergency response efforts in terms of population outreach, routes for emergency resource allocation, or procurement and distribution of medications and other medical supplies. Such an infrastructure ensures ongoing presence of public health workers in regions susceptible to outbreaks, thus helping to speed up access to vulnerable populations in the event of a health security emergency. Information platforms for NCD monitoring and surveillance can also enable detection and response to sequelae of infectious diseases, including developmental, cardiac, neurologic, or pulmonary complications. For example, most patients in Central and South America with chronic Chagas disease, caused by the parasite *Trypanosoma cruzi*, show development of a dilated cardiomyopathy that can be tracked along with other noninfectious causes to determine spikes in transmission.

### NCD Initiatives as a Means for Reducing Susceptibility to Spread of Infections

The presence of clinical links between NCDs and communicable diseases implies that the optimal path to communicable disease control may require consideration of NCDs. The proliferation of NCD risk factors associated with current demographic trends in longevity and urbanization can raise a population’s baseline susceptibility to infection-related health security risks. The detrimental effect of uncontrolled NCDs and NCD risk factors on health security concerns can be illustrated by the examples of diabetes and tobacco use. Diabetes has been shown to increase the severity of endemic diseases such as tuberculosis, melioidosis, dengue, and malaria (*7*–*9*). Diabetes also interferes with tuberculosis treatment, threatening the progress of global tuberculosis control in countries with high rates of both illnesses, such as China and India (*10*,*11*).

Tobacco smoking, besides playing a primary role in all leading NCDs, is a notable risk factor for the acquisition and accelerated progression of variety of infectious diseases, including influenza, tuberculosis, pneumonia, sexually transmitted diseases, and hospital-acquired infections (*12*–*15*). Tobacco use is also among the factors facilitating the convergence of infectious and chronic illnesses in LMICs and compounds the role of other factors, such as urbanization and displacement, in worsening health outcomes. Urbanization in China and India, in particular, has resulted in large groups of rural migrants who are increasingly affected by lifestyle-associated chronic conditions such as diabetes and hypertension while also experiencing added exposure to communicable diseases associated with overcrowding, notably tuberculosis (*13*,*16*). In these examples, focusing on infectious conditions alone without also addressing NCD factors may undermine the principal intent of global efforts to strengthen population health security. Benefits to integration of services across communicable diseases and NCDs have already been shown in the context of treatment for HIV alongside several other chronic conditions (*17*,*18*).

### NCD Initiatives as a Means for Strengthening Economic and Social Outcomes

The economic burden of unaddressed NCDs in developing countries can impair global health security efforts by adding strain on developing economies. More than 80% of NCD-related deaths now occur in LMICs, and NCDs are no longer considered diseases of the developed world (*19*). Because NCD-associated illness in developing countries is more likely to occur prematurely (in persons <70 years of age), illness can be a substantial impediment to human and economic development (*20*,*21*). Reduction in premature NCD deaths in LMICs has been identified as a main goal in the United Nations Agenda for Sustainable Development (*22*). The World Economic Forum estimates that future NCD growth trends could cost the global economy US $47 trillion in cumulative losses through 2030 (*23*). Investment in efforts to reduce NCD-related productivity losses can indirectly reinforce global health security efforts by relieving socioeconomic pressures in some populations and reducing incentives for cross-border migration.

Despite the surge of premature deaths from NCDs in LMICs, resources committed for NCDs in LMICs remain relatively limited (*24*,*25*). Only 1.5% of the development assistance for health distributed to developing countries in 2013 was for NCDs, even as NCDs account for more than half of the all-cause death and disability burden in these countries (*26*,*27*). Fortunately, several cost-effective solutions can make a difference for NCD control in low-resource settings. One option is the standardization of hypertension treatment, recently outlined by the Global Hearts Initiative (*28*), which can simplify and thus enable the broad adoption of treatment protocols for preventing and reducing cardiovascular disease (CVD). CVD, the largest contributor to NCD death and disability in LMICs, may also be addressed in a low-cost manner by exploring the use of generic versions of fixed-dose combination medications, also known as polypills (*4*). Existing laboratory investments can be leveraged relatively easily across both NCD and infectious disease testing, and disease surveillance programs can incorporate NCD monitoring elements. These strategies for NCD control can prove valuable in supporting the goals of health emergency preparedness efforts by strengthening population outreach, patterns for medical resource allocation, and baseline population health.

## Centers for Disease Control and Prevention Initiatives for NCD Prevention and Control

The Global NCDs, Injury, and Environmental Health program with the US Centers for Disease Control and Prevention (CDC) advances a 3-pronged approach to prevention and control by strengthening surveillance, expanding the evidence base, and enhancing workforce capacity (Table 1). The program objectives support the UN Sustainable Development Goals (*29*) and the Global NCD Monitoring Framework (*30*) through supporting training and technical exchange with countries for health promotion activities; using public health data to research innovative, culturally appropriate solutions and improve policy decisions; and supporting the implementation of cost-effective interventions to reduce risk factors such as tobacco use, harmful use of alcohol, unhealthy diets, and physical inactivity. The following programs are some examples of activities that share the long-term goal of contributing to NCD burden reduction in LMICs.

**Table 1:** US Centers for Disease Control and Prevention approaches to NCD, injury, and environmental health control and prevention*

Strategy/activity domain	Goal	Activities	Programs
Strengthening surveillance	Strengthen country and partner capacity for surveillance and monitoring and evaluation systems	• Support surveillance systems through surveys • Use technology to improve data collection, analysis, and reporting • Develop data analysis, dissemination, and visualization tools to track progress toward global NCD targets and evaluate policy impact • Strengthen civil registration, vital statistics, and cause of death and disease registries to inform public health and medical decisions	• Cancer registries • Bloomberg Data for Health Initiative • Global School Health Surveillance • Road traffic injury • Tobacco control • Violence against children
Expanding the evidence base	Scale up interventions to improve health outcomes	• Generate scientific evidence by developing, implementing, and scaling up interventions to accelerate impact for priority risk factors or disease outcomes	• Cervical cancer • Diabetes • Economics of NCD risk factors • Environmental health • Global Hearts Initiative • Maternal mortality • Malnutrition • Shandong Ministry of Health Action on Salt Reduction and Hypertension
Enhancing workforce capacity	Strengthen national public health capacity, infrastructure, and workforce	• Develop training modules • Provide quality training, technical exchange, and mentorship • Utilize web-based training tools • Support mini-grants for relevant projects • Encourage networking	• Field Epidemiology Training Program • NCD short course for program managers

*NCD, noncommunicable disease.

### Standardized Hypertension Management

The Standardized Hypertension Treatment and Prevention project promotes the use of the following evidence-based tools and practices: 1) standardized treatment protocols, 2) team-based care, 3) access to a core set of medications, 4) registries for patient monitoring, 5) patient empowerment, 6) community engagement, 7) policy interventions, and 8) sodium reduction counseling. This project is being piloted in 2 countries. In Barbados, the focus is to improve patient care in 2 publicly funded clinics; in Malawi, the project is designed to enhance 2 HIV clinics funded by the US President’s Emergency Plan for AIDS Relief.

### Global Hearts Initiative

To support governments in strengthening CVD prevention and control, the World Health Organization (WHO), CDC, and other partners launched the Global Hearts Initiative to promote a set of evidence-based interventions that, when used together, can have a major impact on improving global heart health. These interventions include prevention approaches for tobacco control and standardized protocols for CVD management at the primary healthcare level (*31*).

### Prevention and Control of Tobacco Use

The CDC Global Tobacco Control program works with in-country and global partners to monitor the global tobacco epidemic through surveillance systems aimed to assess tobacco use among adults and adolescents to promote tobacco control efforts. CDC provides technical assistance and training packages on tools for standardized surveillance of tobacco use across multiple countries.

### Field Epidemiology Training Programs

Field Epidemiology Training Programs (FETPs) are country-owned programs that strengthen national capacity in epidemiology, surveillance, and outbreak response, including those related to NCDs. Through dedicated curriculum and mentorship, FETP has helped develop expertise within ministries of health in chronic disease surveillance and response, including cardiovascular disease, toxicology, nutrition, tobacco, cancer, injury, and maternal and child health/birth defects. Initial surveillance efforts to first detect and then confirm a causal link between Zika infection and Guillain-Barré syndrome and microcephaly through cohort and case-control studies were led by FETP residents and graduates in Brazil and Colombia. In other locations, FETP residents have led investigations into risk factors for virus-related cancers (e.g., human papillomavirus, hepatitis B virus); current practices related to cervical cancer screening; and the interplay between chronic and infectious disease, such as smoking and tuberculosis. FETP work in nutrition has assisted in emergency famine response, preventing infectious disease outbreaks in displaced persons camps.

### Bloomberg Data for Health Initiative

The goals of this program are to assess the feasibility, quality, and validity of nationally representative mobile phone surveys; implement NCD mobile phone surveys in 10 countries and support face-to-face WHO STEPwise Approach to Surveillance Surveys in 6 overlapping countries; and compare findings from the 2 data collection methods. These surveys will be implemented by participating countries and ministries of health in collaboration with relevant ministries of information and technology, national statistical offices, and telecommunication operators. A course to improve the use of locally available data, entitled Data to Policy, is providing skills in economic evaluation, burden measurement, and impact modeling to develop policy briefs, covering both infectious (e.g., avian influenza, antimicrobial resistance, malaria control) and noninfectious (e.g., colon cancer, tobacco, nutrition) topics.

### Cancer Registries

CDC is working with the International Agency for Research on Cancer of the WHO and other partners to establish 6 regional support centers (hubs) that provide training and assistance to cancer registries around the world. CDC supports these centers in Asia and sub-Saharan Africa and is working with partners to develop a regional hub in the Caribbean. Because little is known about the costs of setting up cancer registries in LMICs, CDC has piloted a cost assessment tool in many of its partner countries. The tool estimates the resources required to operate and improve cancer registries, including funding for the registries, how much it costs to register a cancer case, and factors that affect the efficiency of cancer registries (*32*). The goal is to create accessible information that would help public health leaders to make appropriate decisions on adding registries to their national cancer plans and improve existing cancer registries all over the world (*33*).

### Sodium Reduction and Hypertension

In an innovative partnership, the CDC is working with China’s National Health and Family Planning Commission and the Shandong provincial government on the Shandong Ministry of Health Action on Salt Reduction and Hypertension (SMASH) project (*34*). The aim of SMASH was to reduce daily salt intake from condiments from 12.5 g/day in 2011 to 10 g/day in 2015 and to improve hypertension control within the province. Approaches to reducing sodium intake include changes to food labeling, distribution of scaled spoons for home cooking and preparation, and reforming food industry practices, all of which are being broadly adopted. SMASH also works with restaurants to develop sodium standards for Shandong cuisine, conducts chef training to develop lower-salt menus, tracks salt usage, conducts chef contests for new recipes, and develops communication materials and activities for consumers.

### Micronutrient Malnutrition

Since 2000, the International Micronutrient Malnutrition Prevention and Control Program works with global partners to eliminate vitamin and mineral deficiencies among vulnerable populations throughout the world. This program supports monitoring for micronutrient deficiencies and supports efforts by governments, food industries, and civic organizations to implement interventions, such as food fortification and supplementation. CDC recommends the use of micronutrient powders—sachets of vitamins and minerals that can be mixed into food (home fortification)—to reduce micronutrient deficiencies among children >6 months of age.

## Partnerships of NCD Programs and Global Health Security Agenda Activities

The Global Health Security Agenda (GHSA) (https://www.ghsagenda.org/) is an international effort to prioritize action for global health security. It aims to support the goals of the 2005 International Health Regulations, which strives to increase country capacity for addressing health threats including but not limited to those of infectious origin (*35*). Integration of NCD-related activities into broader disease-control engagements may allow for economies of scale with respect to overall health outcomes. CDC’s Global NCDs, Injury and Environmental Health programs reach ≈40 countries, with potential to address both infectious and noninfectious diseases by linking activities in surveillance, evidence generation, capacity strengthening and partnerships with current GHSA Action Packages (*36*). These programs are organized under 3 broad categories for disease control: prevent, detect, and respond (Table 2).

**Table 2:** Opportunities to incorporate NCD activities within GHSA action packages*

GHSA category	GHSA Action Package	NCD-related activities in support of GHSA goals
Prevent	Immunization	• Human papillomavirus vaccination • Hepatitis B virus vaccination
Detect	National Laboratory System	• Assist laboratories in integrating essential NCD testing into current systems • Train laboratory staff on essential NCD testing
	Real-Time Surveillance	• Integrate NCD indicators into current surveillance systems • Support adoption of EMR • Train staff on EMR use and NCD indicator data entry • Implement monitoring aspects from Hearts Technical Package • Implement Data for Health • Support cancer registries • Support tobacco use surveillance • Enhance birth defects surveillance for Zika virus
	Workforce Development	• Expand NCD training via country-level Field Epidemiology Training Programs • Cross-train local public health staff on NCD basics to link to current efforts, depending on local needs and capacity • Implement workforce training aspects from Hearts Technical Package
Respond	Medical Countermeasures and Personnel Deployment	• Incorporate NCD treatment into public health emergency responses, as appropriate (e.g., natural disasters, refugee crisis, migration)

*EMR, electronic medical records; GHSA, Global Health Security Agenda; NCD, noncommunicable disease.

The relevance of current NCD-related activities to GHSA goals can be illustrated by the potential contributions of ongoing programs such as the Global Hearts Initiative, FETP, and Data for Health. Global Hearts can serve GHSA objectives of threat detection by establishing a mechanism for real-time surveillance and medical workforce development and can support avenues for emergency response by strengthening medication supply chains. Alternative survey methods, such as the mobile phone–based Data for Health initiative, can provide insight into the possibility of using innovative approaches to disease surveillance and detection, especially in low-resource environments where traditional surveillance methods might be too slow or expensive. FETPs can enhance local detection capabilities by training district, regional, and national medical and surveillance personnel in recognizing and preventing emerging threats to health security alongside noncommunicable conditions.

## Conclusions

Incorporating NCD control strategies alongside and within ongoing GHSA efforts for infectious disease control in LMICs can offer a long-term path for a resilient health infrastructure that can respond well both in normal times and during health emergencies. Specific GHSA goals in developing countries can be strengthened by investment in NCD programs like Global Hearts, which can bolster pathways for population outreach, and FETP, which can produce cadres of public health workers.

The growing epidemiologic and infrastructural overlap between NCDs and infectious diseases has motivated increased consideration of NCDs as a component of global health security. Most recently, global resources for NCD prevention and care have increased (*24*) alongside growing recognition that investment in the prevention and control of chronic conditions can improve the capacity to respond to both acute public health crises and long-term health events. To the advantage of NCD control efforts, common NCD challenges in developing countries can be met at relatively modest cost in several ways, from population-level approaches for the prevention of known NCD risk factors like tobacco use to patient-level approaches for low-cost treatment of highly prevalent but treatable conditions like hypertension (*4*,*37*). The synergies between communicable and noncommunicable disease control offer broad implications for developing countries, where building up clinical, laboratory, and regulatory capacity for handling both NCD and emerging disease threats in LMICs can go a long way in improving health security locally and, by extension, worldwide.
